# The microbiota of avocado floral nectar inhibits pathogens and improves plant fitness

**DOI:** 10.1093/jxb/erag038

**Published:** 2026-01-23

**Authors:** Claudia Marina López-García, Indira Aranza Rodríguez-Gómez, Yareli Pérez-Bautista, Luis Alberto Villanueva-Espino, Mariana Molina Torres, Violeta Patiño-Conde, Luis Enrique Ruiz-Guizar, Mariel García-Meléndez, Orlando Hernández-Cristóbal, Jesús Llanderal-Mendoza, Mauricio Quesada, Frédérique Reverchon, Ken Oyama, Alfonso Méndez-Bravo

**Affiliations:** Laboratorio Nacional de Análisis y Síntesis Ecológica, Escuela Nacional de Estudios Superiores Unidad Morelia, Universidad Nacional Autónoma de México, Morelia, Michoacán, México; Laboratorio Nacional de Análisis y Síntesis Ecológica, Escuela Nacional de Estudios Superiores Unidad Morelia, Universidad Nacional Autónoma de México, Morelia, Michoacán, México; Facultad de Biología, Universidad Michoacana de San Nicolás de Hidalgo, Morelia, Michoacán, México; Laboratorio Nacional de Análisis y Síntesis Ecológica, Escuela Nacional de Estudios Superiores Unidad Morelia, Universidad Nacional Autónoma de México, Morelia, Michoacán, México; Laboratorio Nacional de Análisis y Síntesis Ecológica, Escuela Nacional de Estudios Superiores Unidad Morelia, Universidad Nacional Autónoma de México, Morelia, Michoacán, México; Posgrado en Ciencias Biológicas, Ciudad Universitaria, Ciudad de México, México; Escuela Nacional de Estudios Superiores Unidad Morelia, Universidad Nacional Autónoma de México, Morelia, Michoacán, México; Posgrado en Ciencias e Ingeniería en Materiales, Instituto de Física, Ciudad Universitaria, Ciudad de México, México; Laboratorio Nacional de Análisis y Síntesis Ecológica, Escuela Nacional de Estudios Superiores Unidad Morelia, Universidad Nacional Autónoma de México, Morelia, Michoacán, México; Laboratorio Nacional de Análisis y Síntesis Ecológica, Escuela Nacional de Estudios Superiores Unidad Morelia, Universidad Nacional Autónoma de México, Morelia, Michoacán, México; Red de Diversidad Biológica del Occidente Mexicano, Centro Regional del Bajío, Instituto de Ecología, A.C., Pátzcuaro, Michoacán, México; Escuela Nacional de Estudios Superiores Unidad Morelia, Universidad Nacional Autónoma de México, Morelia, Michoacán, México; Escuela Nacional de Estudios Superiores Unidad Morelia, Universidad Nacional Autónoma de México, Morelia, Michoacán, México; Laboratorio Nacional de Análisis y Síntesis Ecológica, Escuela Nacional de Estudios Superiores Unidad Morelia, Universidad Nacional Autónoma de México, Morelia, Michoacán, México; Instituto de Investigaciones en Ecosistemas y Sustentabilidad, Universidad Nacional Autónoma de México, Morelia, Michoacán, México; Red de Diversidad Biológica del Occidente Mexicano, Centro Regional del Bajío, Instituto de Ecología, A.C., Pátzcuaro, Michoacán, México; Escuela Nacional de Estudios Superiores Unidad Morelia, Universidad Nacional Autónoma de México, Morelia, Michoacán, México; Laboratorio Nacional de Análisis y Síntesis Ecológica, Escuela Nacional de Estudios Superiores Unidad Morelia, Universidad Nacional Autónoma de México, Morelia, Michoacán, México; SECIHTI, Ciudad de México, México; University of Cambridge, UK

**Keywords:** *Apis mellifera*, *Colletotrichum gloeosporioides*, floral nectar microbiota, jasmonic acid, *Phytophthora cinnamomi*, plant growth-promoting microorganisms

## Abstract

Floral nectar-living microbes contribute to flower protection and pollinator health and are primarily determined by nectar chemical composition. Microbial communities in non-hexose-rich nectars and their ability to inhibit pathogens and modulate plant development have been poorly explored. We used metabarcoding to examine the richness and relative abundance of bacteria and fungi from avocado, a globally important crop with a unique nectar chemical composition, whose production is severely affected by diseases and low pollination rates. We also explored the antagonistic activity of the nectar microbial culturable fraction and its volatile organic compounds (VOCs) against avocado pathogens *Phytophthora cinnamomi* and *Colletotrichum gloeosporioides*, and against the most devastating honeybee pathogens *Ascosphaera apis* and *Paenibacillus larvae*. Furthermore, we experimentally analyzed the effects of microbial isolates and their VOCs on plant growth and the activation of jasmonic acid (JA) defense responses in *Arabidopsis thaliana*. *Pseudomonas*, *Acinetobacter*, *Protomyces*, and *Vishniacozyma* were the dominant microbial genera inhabiting avocado nectar. From 43 evaluated isolates, 17 bacteria and three yeasts inhibited the plant and honeybee pathogens, promoted the growth of *A. thaliana* seedlings, and induced JA signaling. Microbial VOCs emitted by all tested isolates promoted lateral root formation and increased plant biomass. Collectively, our findings highlight the selectivity of avocado nectar over its microbiota, which could directly impact plant fitness and contribute to the health of its pollinators.

## Teaser

The floral nectar in avocado hosts microbes with anti-pathogenic activity and probiotic properties in a model plant species.

## Introduction

Pollination in angiosperms is a crucial event for the conservation of terrestrial ecosystems that involves a multipartite ecological interaction between flowers, pollinators, and their associated microorganisms (Vannette, 2020). Nectar-consuming insects shape the composition of microbial communities in flowers, which could be neutral, beneficial, or pathogenic for plants (Mueller *et al*., 2023). In turn, the floral microbiota alters nectar chemistry and influence pollinator and plant health (Cullen *et al*., 2021; Martin *et al*., 2022). Nectar, a substance present in flowers, mainly contains sugars and secondary metabolites, and can be considered a niche where only microorganisms tolerant to osmotic stress and low nitrogen availability can develop (Warren *et al*., 2020). Nectar microbial communities tend to be species poor and dominated by a few bacteria and yeast taxa (Mueller *et al*., 2023). Microbial isolates present in nectar can range from plant, pollinator, and even human pathogens, to commensals and attractors of pollinators, and natural enemies of pest insects and phytopathogens (Sasu *et al*., 2010; Adler *et al*., 2021; Álvarez-Pérez *et al*., 2024), positioning nectar as a potential source of beneficial microorganisms for the plant-pollinator interactions and inhibition of plant pathogens. Although some microorganisms isolated from nectar from different flowering crops such as apple, pear, blueberry, and almond trees have been described to inhibit plant pathogens or tolerate different management practices (Smessaert *et al*., 2019; Rering *et al.,* 2023; Schaeffer *et al*., 2023), data on entomopathogenic and/or growth-promoting microorganisms from nectar are still scarce.

*Persea americana* Mill. (avocado) (*Lauraceae*), an ancient angiosperm native of Mexico and Central America, has gained economic relevance worldwide due to its nutritional value (Rendón-Anaya *et al*., 2019; Bhore *et al*., 2021). The market research and consulting firm Grand View Research valued the global avocado market at US$15.83 billion in 2023 (Grand View Research, 2023). However, the agricultural management of this crop is highly input-intensive due to soil impoverishment, incidence of diseases, a lengthy period of vegetative growth required to reach the productive stage, and low pollination rates (Pérez-Jiménez, 2008; Twizeyimana *et al*., 2013; Santini *et al.*, 2022). Root rot caused by the soil-borne oomycete *Phytophthora cinnamomi* is the most serious disease, with a decrease in production of between 45% and 90% in affected areas (Pérez-Jiménez, 2008), while anthracnose caused by the opportunistic phytopathogen *Colletotrichum gloeosporioides* is the main cause of post-harvest losses (Xoca-Orozco *et al*., 2017). Over the past 5 years, major avocado-producing countries have reported phytosanitary alerts due to *C. gloeosporioides* incidence in orchards (Ayvar-Serna *et al*., 2021; Bustamante *et al*., 2022), affecting up to 80% of production with significant economic losses (Fuentes-Aragón *et al*., 2020; Bernal *et al*., 2025). Although anthracnose is a disease that manifests in fruits, infection usually occurs at the flowering stage. As flowers may host bacteria and fungi that inhibit the growth of several *Colletotrichum* spp. pathogens, as described in blueberries (Rering *et al*., 2023), the potential of nectar microorganisms to act as biocontrol agents of *C. gloeosporioides* in avocado should be investigated. On the other hand, previous studies have shown that certain volatile organic compounds (VOCs) produced by microorganisms associated with avocado antagonize the growth of *P. cinnamomi* (Guevara-Avendaño *et al*., 2019), which highlights the relevance of investigating the ability of VOCs produced by avocado nectar-living microorganisms to suppress the growth of this oomycete.

The small, greenish avocado flowers produce a peculiar nectar with a high sucrose concentration (79–80%), a small fraction of fructose (1%), and traces of K^+^, stachyose, and perseitol (Liu *et al*., 1995). Flowers open twice; first in the pistillate female phase and then in the male staminate phase (Bender, 2002; Can-Alonzo *et al*., 2005). This dichogamous flowering pattern, combined with the low pollen release and highly specific chemical composition of avocado nectar, leads to <1% of the flowers reaching fruit set (Bender, 2002), the remainder falling to the ground and decomposing, probably incorporating their microbiota into the soil. One of the strategies implemented to remedy the low pollination rate in avocado has been the introduction of *Apis mellifera*, positively impacting the production in commercial orchards (Peña and Carabalí, 2018). Unfortunately, the management of this generalist pollinator has caused the emergence of pathogens such as the bacterium *Paenibacillus larvae* (Fünfhaus *et al*., 2018) and the fungus *Ascosphaera apis* (Chaimanee *et al*., 2017), leading to bee colony losses (Genersch, 2010; Goulson *et al*., 2015). In this context, it is important to determine whether *P. larvae* or *A. apis* is part of the avocado nectar microbiota or whether nectar microorganisms could antagonize the growth of these bee pathogens.

The high selectivity of floral nectar as a microenvironment and the resulting competitiveness of its microbiota (Warren *et al*., 2020; Mueller *et al*., 2023), combined with the close contact between nectar-living microorganisms and plant tissues (Sasu *et al*., 2010), led us to hypothesize that (i) avocado nectar, with its particular chemical composition, recruits a different microbial community from that described in other plant models; (ii) the nectar microbiota performs probiotic functions in their plant hosts and pollinators as antagonists of pathogens; and (iii) the microbiota possesses phytostimulating properties influencing endogenous plant signaling. Our goal was to find broad-spectrum antagonistic strains, active against both phytopathogens and entomopathogens, capable of activating molecular signaling in host plants. We used metabarcoding sequencing to explore the diversity and composition of the avocado nectar microbial community and a culture-dependent approach to characterize the antagonistic activity of avocado nectar microbiota against the avocado pathogens *C. gloeosporioides* and *P. cinnamomi* and the honeybee pathogens *A. apis* and *P. larvae.* Moreover, we tested the ability of nectar-inhabiting microorganisms to promote plant development and defense in the model plant *Arabidopsis thaliana*. Collectively, this information could help to eventually design sustainable strategies to mitigate the impact of diseases that affect avocado production, whilst contributing to understand multipartite interactions between pollinators, plants, and their associated microbiota.

## Materials and methods

### Study site and sampling

The study site was located at Acuitzio del Canje, state of Michoacán, Mexico (19°30′N; 101°20′W) at 2080 m a.s.l., in the main avocado-producing area worldwide (FAOSTAT, 2016). The neighboring vegetation corresponds to oak forests. Rainfall ranges between 800 mm and 1300 mm year^−1^, with an average annual temperature of 24 °C, a maximum temperature of 32 °C, and a minimum of 10 °C. Avocado nectar samples were collected with sterile 5 µl capillaries by random sampling of at least 10 female-phase flowers from five different avocado trees without symptoms of any disease in March 2019. Samples were transported to the laboratory for microbiological or molecular processing.

### Metabarcoding and bioinformatic analyses

To characterize the nectar microbial communities, we extracted genomic DNA from nectar samples using the DNeasy Blood and Tissue Kit (QIAGEN), according to the manufacturer’s instructions. Briefly, the nectar was collected from 45 female-stage flowers on five healthy trees. Subsequently, nectar from 15 flowers was pooled to construct three libraries for 16S rDNA and internal transcribed spacer 2 (ITS2) metabarcoding.

Library preparation was performed at the Genomics Lab of the National Laboratory of Ecological and Synthesis (LANASE) following Illumina’s procedure with some modifications. A 5 µl aliquot of each nectar pool was used to characterize the bacterial community by amplifying the V4 region of the 16S rDNA gene, using primers 515-YF (Parada *et al*., 2016) and 806R (Apprill *et al*., 2015) (Supplementary Table S1). For the fungal community, the same amount of nectar from the three different pools was used to amplify the ITS2 region, with a mixture of four forward primers and the degenerate reverse primer ITS4NGS (Tedersoo *et al*., 2014) (Supplementary Table S1). All PCRs had a total volume of 50 μl, containing 5 μl of nectar-extracted DNA, 0.2 μM of each primer, and 25 μl of 1× Qiagen Multiplex PCR Master Mix. The PCR cycling parameters were: 95 °C for 15 min; 25 cycles at 94 °C for 30 s, 55 °C for 30 s, 72 °C during 30 s; and a final extension for 5 min at 72 °C. The amplicons were indexed with the Nextera XT kit v2 and purified with the ProNex® Size Selective Purification System, following the Purification PCR amplicon protocol. Each library was analyzed by capillary electrophoresis with a Qiaxcel system to estimate their final size (450 bp for the 16S rDNA gene and from 500 bp to 620 bp for the ITS2 metabarcode, including indexes and adapters). Paired-end sequencing (2×250 bp) was performed on a MiSeq instrument (Illumina, USA) at the Sequencing Unit of the Mexican National Institute of Genomic Medicine (INMEGEN). Each library was sequenced in triplicate. The generated sequences were demultiplexed and the primers removed. Bioinformatics analysis was conducted in QIIME2-2023.7, using DADA2 (Callahan *et al*., 2016) for trimming and truncating low-quality reads, removing chimeras, and generating amplicon sequence variants (ASVs). Taxonomy assignments were performed with the q2-feature-classifer sklearn with self-trained classifiers (Bokulich *et al*., 2018). For the 16S trained model, the ‘SILVA 138 at 99%’ database (Robeson *et al*., 2021) was restricted based on the primers (Werner *et al*., 2012), and default features were used. The ITS model was trained with full sequences without restriction of the UNITE ‘sh_qiime_release_s_04.04.2024’ database (Abarenkov *et al*., 2024), and a k-mer length of [6,6] (Bokulich *et al*., 2018) and a confidence limitation of the 98% assignments were used. Plastid, mitochondrial, and archaeal reads were removed from 16S assignment, and non-assigned reads at the phylum level were removed for both datasets. Data derived from bacterial and fungal sequencing were deposited in the Sequence Read Archive of NCBI under accession PRJNA1236352 (https://www.ncbi.nlm.nih.gov/sra/PRJNA1236352).

### Isolation of the nectar culturable microbial fraction

Nectar samples from 50 female-stage flowers on five healthy trees were immediately mixed with 100 µl of 0.9% sodium chloride upon arrival at the laboratory. Suspensions were spread on the surface of Petri dishes with solid R2A medium supplemented with 20% sucrose and incubated at 28 °C. Isolated colonies were re-streaked on Luria–Bertani (LB) solid medium and potato dextrose agar (PDA) supplemented with 100 mg l^−1^ ampicillin until pure cultures were obtained. The 97 resulting microbial isolates were grouped into 43 morphotypes, according to morphological criteria and Gram staining as described in Guevara-Avendaño *et al*. (2019). All isolates were preserved at −20 °C in LB or potato dextrose broth supplemented with 20% glycerol.

### Antagonism assays against avocado pathogens

Avocado pathogens were the fruit fungal pathogen *C. gloeosporioides* and the soil-borne oomycete *P. cinnamomi*. To implement the dual antagonism assays, 10 µl of a standardized suspension (0.5 of absorbance at OD_600_) from each nectar microbial isolate were inoculated at 2.5 cm from a mycelium disc (5 mm diameter) placed in the center of PDA plates. Three isolates were evaluated per plate. As a control, 10 µl of LB medium were inoculated, following the design described in Guevara-Avendaño *et al*. (2018). To evaluate the antagonistic activity of strains by volatile emission, the two-sealed-base-plates method described in Guevara-Avendaño *et al*. (2019) was employed. Briefly, the strains were pre-grown for 24 h as described above, but lids were replaced by another base plate containing a disc of 5 mm diameter of fungal or oomycete mycelium on PDA. The plates were sealed on top of each other with Parafilm® and incubated at 28 °C. Three assays were set up only with growth medium as controls. After 5 d of incubation, the fungal growth inhibition percentage was determined by measuring the mycelial diameter, with the formula described in Guevara-Avendaño *et al*. (2018). Each isolate was tested in triplicate. To assess the effect of the antagonistic isolates on the hyphal morphology, 10 representative mycelium samples of each fungal/oomycete phytopathogen grown under control conditions and in the presence of the two isolates with the highest antifungal activity were collected, processed, and analyzed by SEM in a JEOL JSM-IT300 microscope according to Méndez-Bravo *et al*. (2018).

The *in vivo* antifungal activity of the two most active isolates against *C. gloeosporioides* was evaluated by implementing wound and drip assays in anthracnose-free avocado fruits as described by Trinidad-Ángel *et al*. (2017) with modifications. Twenty ripe and healthy Hass fruits were washed and disinfected with 1% sodium hypochlorite for 5 min and rinsed three times with sterile distilled water, followed by immersion in 70% ethanol, then air-dried in a laminar flow hood. After disinfection, four fruits were designated as the control; the other 16 were wounded on two opposite sides in the middle part with a cork borer to generate cavities of 5 mm diameter and 3 mm depth. Wounded fruits were divided into groups of four for the different treatments: (i) 20 µl of sterile water on both sides (mock, negative control); (ii) 20 µl of a pathogen spore solution at a concentration of 1×10^5^ spores ml^−1^ applied on both sides (positive control); (iii) 20 µl of bacterial suspension at a concentration of 1×10^6^ CFU ml^−1^on both sides (biocontrol agent); and (iv) 20 µl of bacterial suspension plus 20 µl of the pathogen spore solution on both sides (pathogen+biocontrol agent). All fruits were placed in plastic containers and incubated for 10 d in a plant growth chamber (Lumistell ICP-55, Mexico) with a photoperiod of 16 h of light, 8 h of darkness, light intensity of 200 μmol m^2^ s^−1^. After the incubation period, fruits were cut in half and photographed to determine the size of the lesions caused by *C. gloeosporioides* using the ImageJ2/Fiji program (Wayne Rasband National Institutes of Health, USA).

### Molecular identification of nectar isolates

Nectar isolates with ≥20% inhibition against *C. gloeosporioides* and *P. cinnamomi* were cultured in liquid medium for 48 h at room temperature and then centrifuged at 3500 *g* for 15 min. The pellets were resuspended in phosphate-buffered saline (PBS), adjusting the biomass to ∼100 mg FW, and DNA was extracted using the Dneasy Blood and Tissue kit (Qiagen, The Netherlands). The genomic DNA concentration was adjusted to 25 ng µl^−1^. To identify bacterial isolates, primers 27F and 1492R were used to amplify the 16S rRNA gene, while for yeasts, primers ITS-1 and ITS-4 were used to amplify the ITS region (Supplementary Table S1). Amplifications were performed in a SureCycler 8800 (Agilent Tech., CA, USA), using 50 µl of reaction mix containing 25 ng of template DNA, 200 µM of each dNTP, 1.25 mM of MgCl_2_, 1× Taq buffer, 0.4 µM of each primer, and 0.5 U of Taq DNA polymerase (Qiagen, Germany). For 16S rRNA amplification, the PCR conditions werte as follows: initial step at 95 °C for 4.5 min; 40 cycles at 95 °C for 1 min, 53 °C for 1 min, and at 72 °C for 2 min; with a final step at 72 °C during 5 min. For amplification of the ITS region, the PCR cycling parameters were 95 °C for 3 min, followed by 35 cycles at 95 °C during 45 s, 56.2 °C for 35 s, 72 °C for 45 s, and a final single cycle at 72 °C for 5 min. Amplicons were purified using the Wizard® SV Gel and PCR Clean-Up System (Promega, USA) and adjusted to 20 ng µl^−1^ for sequencing on a Hitachi 3500 Genetic Analyzer (Applied Biosystems) at ENES-UNAM Morelia. The obtained sequences were deposited in GenBank (accession nos PV366450–PV366466 and PV366592–PV366594). Sequences were manually checked in BioEdit 7.2.5. and aligned using MEGA 7, utilizing the multiple alignment program MUSCLE. The edited sequences and their best matches in the GenBank nucleotide database (www.ncbi.nlm.nih.gov) were used to construct the alignment.

### Antagonism assays against bee pathogens

The strains of *A. apis* and *P. larvae* were acquired at the Mexican National Collection of Microbial Strains and Cell Cultures from the Centro de Investigación y de Estudios Avanzados del Instituto Politécnico Nacional (CINVESTAV).

Antagonism assays against *A. apis* were carried out as described above for the avocado pathogens, using maltose yeast extract (MYM) plates as medium instead of PDA. To assess the antifungal activity of the isolates against *P. larvae*, 200 µl of a standardized suspension (0.5 absorbance at OD_600_) of *P. larvae* were spread on the surface of Petri dishes with Brain Heart Infusion (BHI) agar. Four 5 mm paper discs moistened with 10 µl of standardized suspensions of three nectar microbial isolates, and 10 µl of LB medium as a control, were placed at the cardinal points of each plate. After 5 d of incubation at 28 °C, the halo of inhibition was measured. Each combination of isolates was tested in triplicate. The growth on MYM and BHI agar medium of each isolate was previously corroborated.

### Effect of nectar isolates on plant growth and development

*Arabidopsis thaliana* (L., Heynh) ecotype Columbia 0 (Col-0) and reporter line *JAZ1/TIFY10A::GFP:uidA* (Grunewald *et al*., 2009) were used to evaluate the effect of the selected isolates on plant growth and jasmonic acid (JA) response. Arabidopsis seeds were surface-disinfected with 96% ethanol for 5 min and 20% sodium hypochlorite for 7 min. After five washes with sterile distilled water, seeds were stored at 4 °C during 48 h for stratification. Growth promotion induced by volatiles was tested *in vitro* sowing five seeds per plate, and eight seeds were sown in undivided Petri dishes to evaluate the effect of the isolates in dual culture assays. The plates, which contained solid 0.2× MS medium (Murashige and Skoog basal salts mixture, Cat. M5524; PhytoTechnology), were placed vertically into a plant growth chamber (Lumistell ICP-55, Mexico) with a photoperiod of 16 h of light, 8 h of darkness, light intensity of 200 μmol m^2^ s^−1^, and temperature of 22 °C.

Four days after germination, seedlings in undivided plates were inoculated with 100 µl (10^8^ CFU ml^−1^) of microbial suspension, placed at a distance of 2.5 cm from root tips, to assess the plant growth-promoting effect of nectar isolates. To evaluate the effect of volatiles emitted by nectar isolates, 100 µl (10^8^ CFU ml^−1^) of bacterial suspension were inoculated in the plant-less compartment of the divided Petri plates. Control plants were grown without microbial inoculant. All treatments were performed in triplicate (three different dishes) in three independent experiments (three replicates). Root system architecture (primary root length and lateral root number), biomass accumulation, and *JAZ1* expression were analyzed at 7 d after inoculation. The histochemical analysis of transgenic seedlings to evaluate the expression of *JAZ1-GUS* was performed with the β-glucuronidase Reporter Gene Staining Kit (Sigma-Aldrich), according to the manufacturer’s instructions. Then, plant tissue was clarified according to the protocol of Malamy and Benfey (1997), and seedlings were placed on glass slides, covered with coverslips, and sealed with commercial nail varnish to analyze the *JAZ1* expression with an optical microscope (Zeiss AXIO Zoom V16 with an Axiocam 305 color camera, Germany).

### Statistical analyses

A Shapiro–Wilk test was used to test whether data fitted a normal distribution. A multiple one-way ANOVA with a Tukey’s post-hoc test was conducted using STATISTICA v.10 software to determine statistical differences between treatments, using a *P*-value ≤0.05. Additionally, the treatments in antagonism assays were compared with the control using a post-hoc Dunnett test.

## Results

### Microbial community composition in the avocado floral nectar

**Fig. 1.**
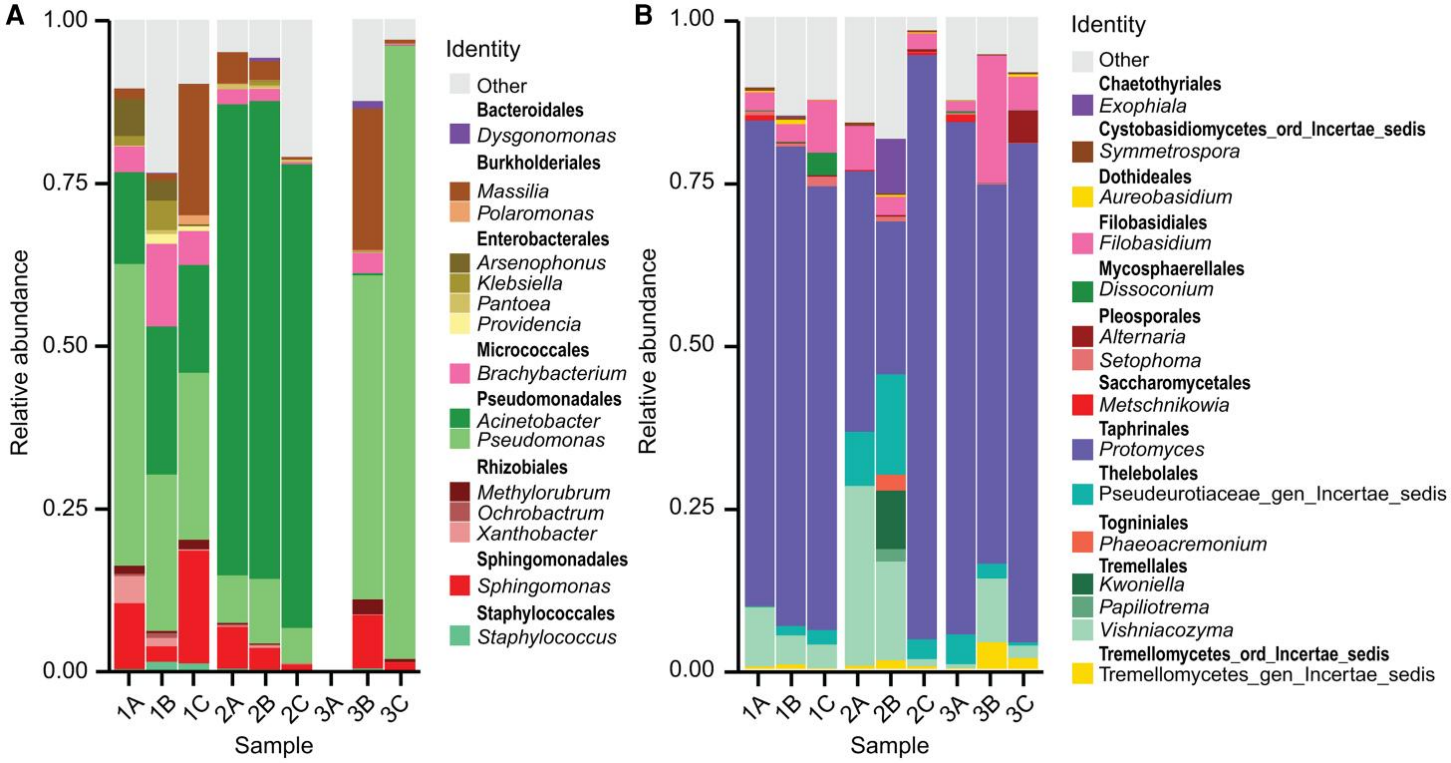
Structure of the microbial community associated with the avocado floral nectar. Relative abundance of (A) bacterial and (B) fungal genera obtained by metabarcoding sequencing. A total of 634 bacterial ASVs with a 99% confidence limitation and 243 fungal ASVs with a 98% confidence limitation were assigned.

We employed a metabarcoding approach to explore the complexity of the nectar microbial community to assess the bacterial and fungal richness and abundance. A total of 634 bacterial ASVs and 249 fungal ASVs, with a minimal size of 234 bp after filtering with DADA2, were recorded. The rarefaction analysis revealed that the abundance of ASVs reached a plateau, indicating that the saturation of sequencing depth for the samples was reached, except for the triplicate 3A in the 16S sequencing. The dominant bacterial order was Pseudomonadales, represented by the *Acinetobacter* and *Pseudomonas* genera, reaching a relative abundance ranging from 40% to 94%, followed by *Massilia* and *Polaromonas* associated with Burkholderiales, and by *Sphingomonas* (Fig. 1A; Supplementary Fig. S1). The most abundant fungal ASVs were *Protomyces* (23–89%), the phyllosphere-inhabiting *Vishniacozyma* (0.6–27%), and *Filobasidium* (1.5–19.5%) (Fig. 1B; Supplementary Fig. S2). The low diversity and large dominance recorded for the bacterial and fungal communities from nectar samples provide evidence of the high selectivity that avocado nectar exerts over the microbial community.

### Strains isolated from avocado floral nectar differentially inhibit the growth of *C. gloeosporioides* and *P. cinnamomi*

**Fig. 2.**
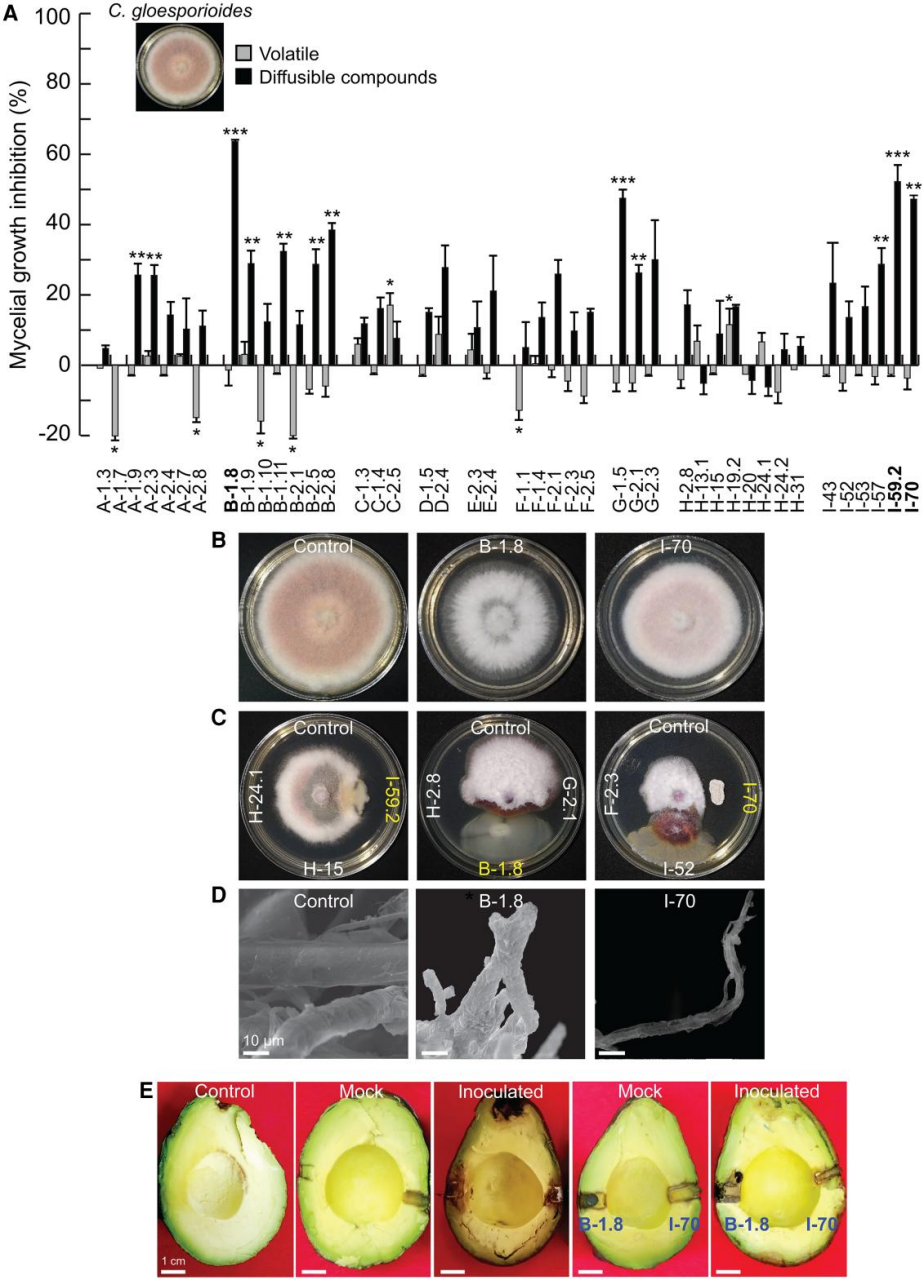
Antagonistic effect of selected microbial isolates from avocado floral nectar against *Colletotrichum gloeosporioides*. Antifungal activity of volatile and diffusible compounds produced by 43 representative morphotypes was evaluated under *in vitro* conditions, and the most active of them were tested in avocado fruit rot inhibition assays. (A) Percentage of inhibition of mycelial growth by emission of volatile and diffusible compounds. (B) Representative images of the mycelial growth inhibition of plugs of *C. gloeosporioides* exposed to volatiles emitted by the most active microbial isolates. (C) Images of mycelium in dual culture inhibition assays with the most active isolates. (D) SEM images of the morphological alterations in the hyphae structure induced by the isolates. (E) Representative images of the avocado fruit rot inhibition assays. The values represent the mean ±SE (*n*=3) and asterisks indicate a significant difference compared with control (Dunnett test, *P*≤0.05).

To explore the potential function of bacteria and fungi recorded by the metabarcode, we obtained a culturable fraction isolated from the avocado floral nectar. A total of 50 flowers from five avocado trees were sampled in the morning immediately after anthesis, avoiding the arrival of nectar-consuming insects. From the nectar of these 50 flowers, 97 isolates were obtained, which were classified into 43 morphotypes (Supplementary Table S2); 51% of them were Gram-positive bacteria, 39% Gram-negative bacteria, and 9% yeasts. Randomly selected representative strains of each morphotype were used to perform antagonism assays against *C. gloeosporioides* and *P. cinnamomi* by volatile emission or dual culture assays. Two isolates (C-2.5 and H-19.2) showed significant inhibition of the mycelial growth of *C. gloeosporioides* through volatile emission (Fig. 2A), while 17 isolates inhibited radial growth in dual culture assays (Fig. 2A). Isolates I-59.2, B-1.8, and I-70 showed the highest antagonistic activity in dual culture assays and, although their inhibitory effect through the emission of volatiles was not significant, B-1.8 and I-70 caused a lower mycelial growth density by indirect interaction (Fig. 2B, C). These two isolates were then selected to evaluate their effect on hyphae morphology and to perform an avocado fruit rot inhibition assay. SEM micrographs of *C. gloeosporioides* mycelium grown in the vicinity of B-1.8 showed that this strain decreased the thickness of hyphae, causing their collapse and abnormal branching, while I-70 caused hyphal collapse (Fig. 2D). The confrontation of B-1.8 and I-70 against *C. gloeosporioides* by wound and drip in the exocarp of avocado fruits demonstrated that these isolates reduced the damage caused by anthracnose by ∼50% and 94%, respectively (Fig. 2E; Supplementary Fig. S3).

**Fig. 3.**
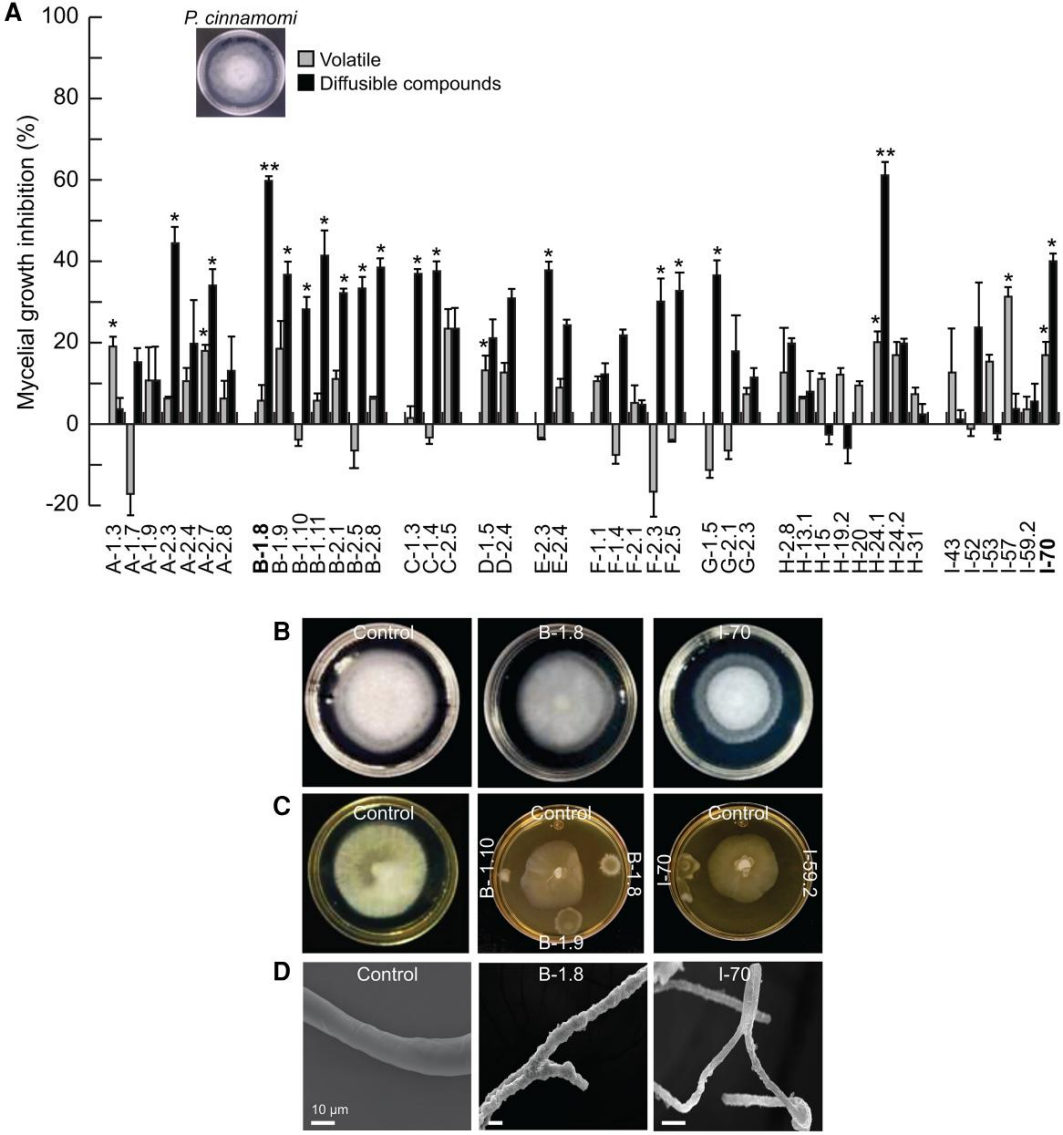
Inhibition of *Phytophthora cinnamomi* growth by strains isolated from avocado floral nectar. (A) Inhibition percentage of mycelial radial growth by emission of volatile and diffusible compounds of the microbial strains. (B) Representative images of inhibition assays by volatile emission or (C) in dual culture assays with the most active strains. (D) SEM images of morphological alterations induced by the isolates with the highest percentage of inhibition. The values shown represent the mean ±SE (*n*=3) and asterisks indicate significant differences with respect to control (Dunnett test, *P*≤0.05).

In the inhibition tests against *P. cinnamomi*, six isolates inhibited mycelium growth by the emission of volatile compounds and 25 of them showed a percentage of inhibition >20% by direct contact (Fig. 3A). The volatile compounds from five isolated strains, again including the one identified as I-70, reduced the aerial mycelium density of the oomycete. Although strain B-1.8 did not inhibit this phytopathogen through volatile compounds on this occasion, it was the most effective of 17 bioactive isolates against *P. cinnamomi* in dual culture assays (Fig. 3A, B). SEM micrographs showed a drastic reduction in hyphal thickness caused by B-1.8, whilst I.70 induced hyphal thinning and distortion. Both isolates caused shriveling of the hyphal surface (Fig. 3D).

### Molecular identification of avocado nectar strains antagonistic to *C. gloeosporioides* and *P. cinnamomi*

The 20 isolates (17 bacteria and 3 yeasts) that were active against the phytopathogens were tentatively identified by 16S rRNA and rDNA–ITS1–ITS4 gene sequencing. The closest sequence matches based on the BLAST similarity analysis are shown in Table 1. The most represented bacterial genus was *Pseudomonas* with 10 isolates that showed 99–100% identity, being closely related to *P. flavescens* (A-1.9 and A-2.7), *P. aylmerensis* (A-2.3, B-1.8 and F-2.5), *P. protegens* (B-1.9), *P. cerasi* (B-1.10 and B-2.5), and *P. viridiflava* (B-1.1 and G-2.1). Seven of the remaining isolates were closely related to *Paenibacillus favisporus* (B-2.1), *Erwinia aphidicola* (B-2.8), *Curtobacterium ammoniigenes* (C-1.3), *Nocardioides endophyticus* (I-57), *Klebsiella quasipneumoniae* (G-1.5), *Streptomyces eurythermus* (I-70), and *Dietzia timorensis* (I-59.2). The best match for the ITS1 and ITS4 sequences classified the three yeast isolates into the *Filobasidium* (C-1.4 and G-2.3) and *Aureobasiduim* (D-1.5) genera (Table 1). The taxonomic identity of the active culturable fraction matches the dominance of the genera recorded with the analysis of the microbial communities.

**Table 1.** Molecular identification of selected isolates from avocado nectar

ID	GenBank accession number	NCBI best match (accession number)	Identity (%)
A-1.9	PV366466	*Pseudomonas flavescens* (NR_114195.1)	98.02
A-2.3	PV366450	*Pseudomonas aylmerensis* (NR_169460.1)	100
A-2.7	PV366465	*Pseudomonas flavescens* (NR_114195.1)	96.02
B-1.8	PV366451	*Pseudomonas aylmerensis* (NR_169460.1)	100
B-1.9	PV366461	*Pseudomonas protegens* (NR_114749.1)	100
B-1.10	PV366457	*Pseudomonas cerasi* (NR_146827.1)	100
B-1.11	PV366463	*Pseudomonas viridiflava* (NR_114482.1)	100
B-2.1	PV366455	*Paenibacillus favisporus* (NR_029071.1)	99.7
B-2.5	PV366459	*Pseudomonas cerasi* (NR_146827.1)	100
B-2.8	PV366453	*Erwinia aphidicola* (NR_117000.1)	100
C-1.3	PV366452	*Curtobacterium ammoniigenes* (NR_041495.1)	100
C-1.4	PV366593	*Filobasidium chernovii* (NR_073223.1)	98.9
D-1.5	PV366594	*Aureobasiduim melanogenum* (NR_159598.1)	97
F-2.5	PV366454	*Pseudomonas aylmerensis* (NR_169460.1)	100
G-1.5	PV366458	*Klebsiella quasipneumoniae* (NR_132596.1)	100
G-2.1	PV366455	*Pseudomonas viridiflava* (NR_114482.1)	99.7
G-2.3	PV366592	*Filobasidium chernovii* (NR_073223.1)	98.9
I-59.2	PV366462	*Dietzia timorensis* (NR_112775.1)	100
I-57	PV366460	*Nocardiodes endophyticus* (NR_135731.1)	100
I-70	PV366464	*Streptomyces eurythermus* (NR_025869.2)	99.6

### Effects of the phytopathogen-antagonistic strains against *Apis mellifera* pathogens

**Fig. 4.**
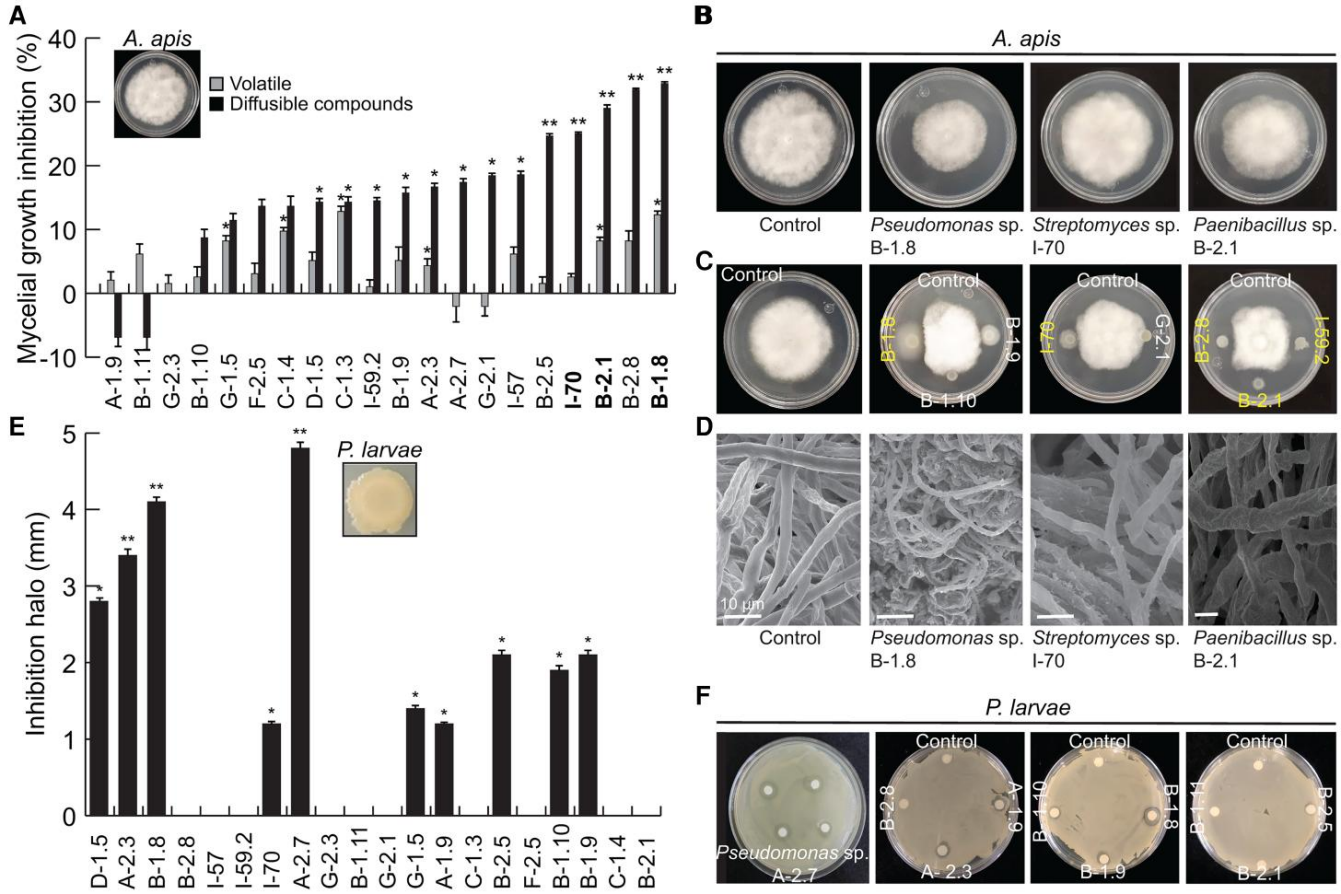
Effect of the identified isolates on the growth of honeybee pathogens. (A) Percentage of inhibition of *Ascosphaera apis* mycelial growth by the emission of volatile and diffusible compounds by the selected microbial strains. (B) Representative images of mycelial growth inhibition of *A. apis* exposed to the volatiles released by the most active strains. (C) Dual culture inhibition assays of *A. apis* with the most active strains. (D) SEM images of the morphological alterations in the hyphal structure of *A. apis* induced by the selected strains. (E) Antibacterial activity of the selected strains co-cultured with *Paenibacillus larvae*. (F) Images of the inhibition assays after 5 d. The values shown represent the mean ±SE (*n*=3) and the asterisk indicates a significant difference relative to control (Dunnett test, *P*≤0.05).

With the aim of exploring how broad the spectrum of the nectar strains to inhibit pathogens would be, not only against phytopathogens but also against entomopathogens, the 20 identified isolates were screened for their potential antagonistic activity against the fungus *A. apis* and the bacterium *P. larvae*. Five bacterial isolates and one yeast (C-1.4) showed moderate but significant activity against *A. apis* (*P*≤0.05) by volatile emission; notably, 13 of the tested isolates displayed antifungal activity by direct contact in the dual culture assays (Fig. 4A). From these 13 active strains, *Pseudomonas* spp. B-2.5 and B-1.8, *Streptomyces* sp. I-70, *Paenibacillus* sp. B-2.1, and *Erwinia* sp. B-2.8 inhibited the pathogen mycelial growth by >20% (Fig. 4A–C). In particular, the isolates I-70, B-2.1, and B-1.8 caused hyphal wrinkling and shriveling on *A. apis* (Fig. 4D).

Ten of the nectar isolates co-cultured with *P. larvae* generated growth inhibition halos ranging from 1 mm to 5 mm. *Pseudomonas* spp. A-2.7, B-1.8, and A-2.3 showed the highest inhibitory activity, followed by the yeast *Aureobasidium* sp. D-1.5 (Fig. 4E, F).

### Plant growth-promoting properties of avocado nectar isolates

**Fig. 5.**
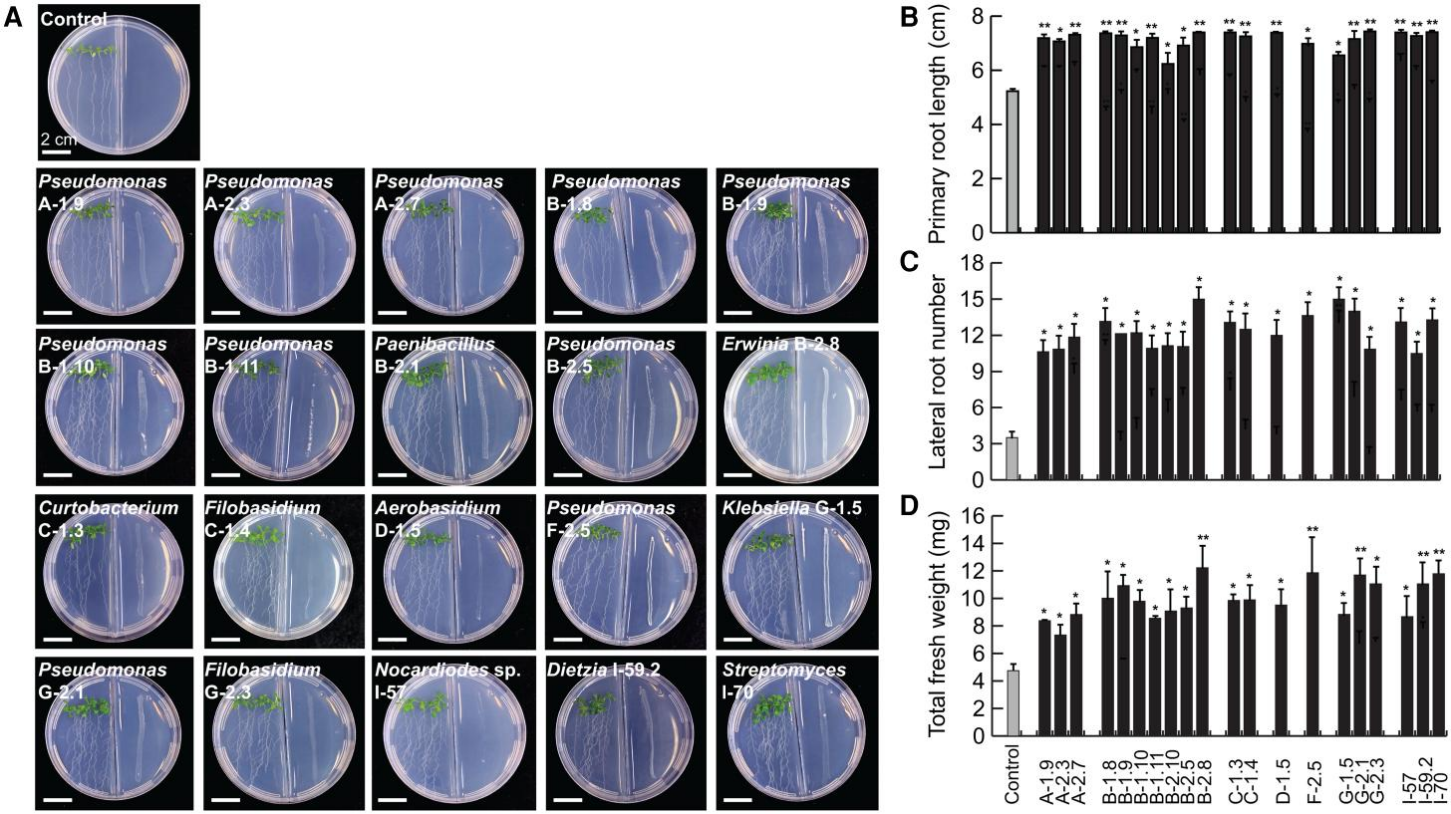
Effects of volatile compounds produced by the isolates from avocado nectar on the root system architecture and biomass accumulation in Arabidopsis. Four-day-old seedlings were exposed for 7 d to the volatiles released by the selected isolates to determine their effect on plant development. (A) Representative images of Arabidopsis seedlings grown in compartmentalized plates in interaction with volatiles produced by the isolates. (B) Quantitative analysis of primary root length, (C) lateral root number, and (D) total FW of seedlings grown on the surface of agar 0.2× MS-containing plates. Values shown represent the mean ±SE (*n*=45 seedlings) and asterisks indicate significant differences between treatments (Tukey test, *P*≤0.05).

**Fig. 6.**
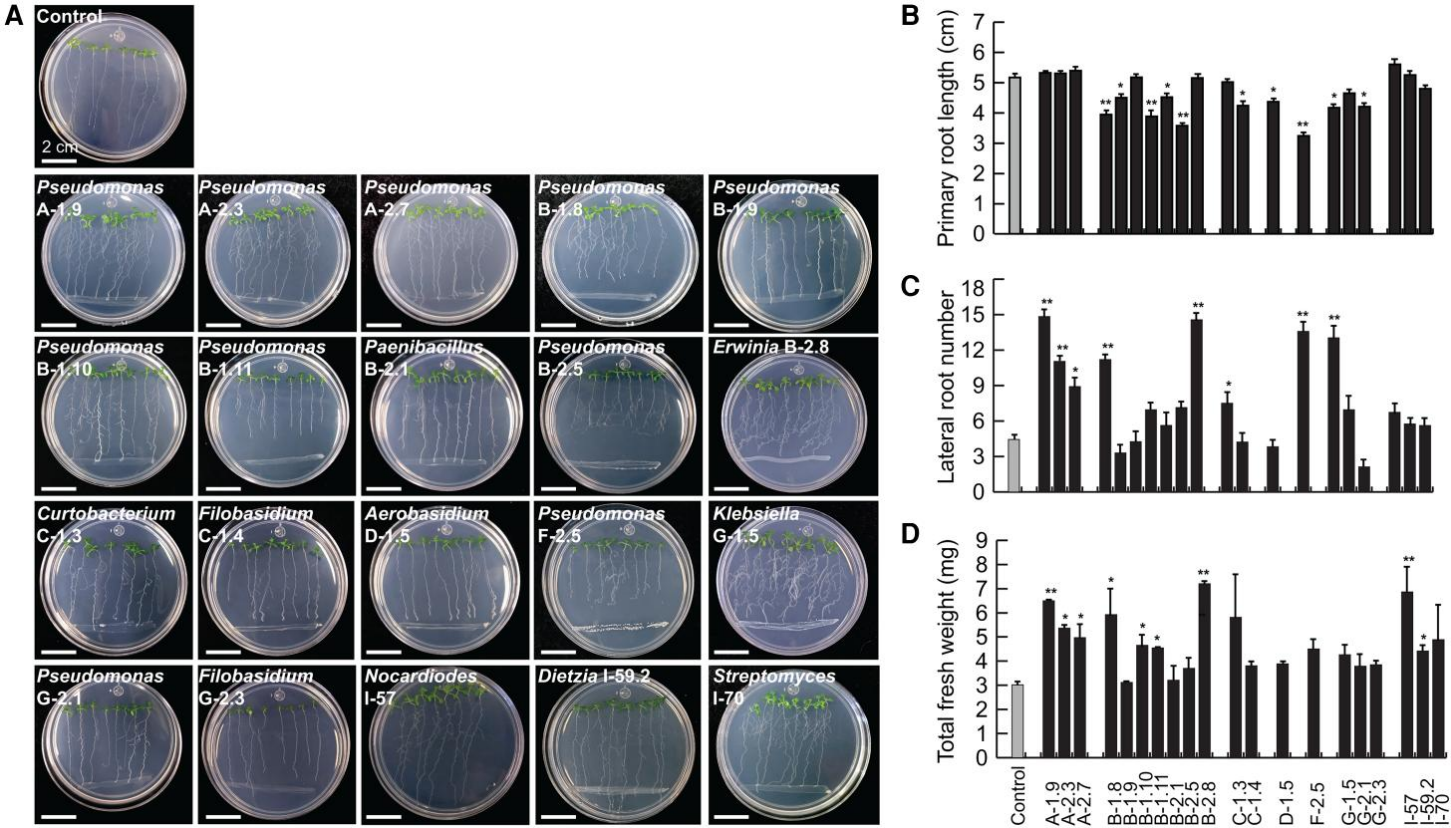
Effects of the co-cultivation of the microbial isolates with Arabidopsis seedlings on root growth and development and biomass accumulation. Four-day-old seedlings were transferred to 0.2× MS medium inoculated with the identified isolates to analyze root architecture and biomass accumulation 7 d after interaction. (A) Representative images of Arabidopsis seedlings interacting with the identified isolates. (B) Quantitative analysis of primary root length, (C) lateral root number, and (D) total FW. Values shown represent the mean ±SE (*n*=72 seedlings) and asterisks indicate statistically significant differences (Tukey test, *P*≤0.05).

As a first approach to explore the potential capacity of the 20 bioactive isolates to establish multipartite interactions, beyond pathogen inhibition, we performed *in vitro* interaction assays with *A. thaliana* to evaluate the microbial effect on plant development and the activation of the induced systemic response. Remarkably, the volatiles emitted by all the microbial isolates increased the primary root length, promoted lateral root organogenesis and growth, and stimulated biomass accumulation of *A. thaliana* seedlings (Fig. 5A–D). When the isolates were co-cultured with the seedlings by direct contact, most of them modified the root architecture; isolates *Pseudomonas* spp. A-1.9, A-2.3, and A-2.7, *Nocardioides* sp. I-57, and *Streptomyces* sp. I-59.2 stimulated root branching or biomass accumulation without any inhibitory effect on primary root length (Fig. 6A–D), while *Pseudomonas* spp. B-1.8 and F-2.5, *Erwinia* sp. B-2.8, *Curtobacterium* sp. C-1.3, and *Klebsiella* sp. G-1.5 decreased the primary root length but stimulated lateral root formation and increased the FW accumulation (Fig. 6A–D). These results indicate that the avocado nectar microorganisms possess plant growth-promoting activity and could differentially impact plant signaling pathways.

### Induction of defense responses in *A. thaliana* by the plant growth-promoting isolates

**Fig. 7.**
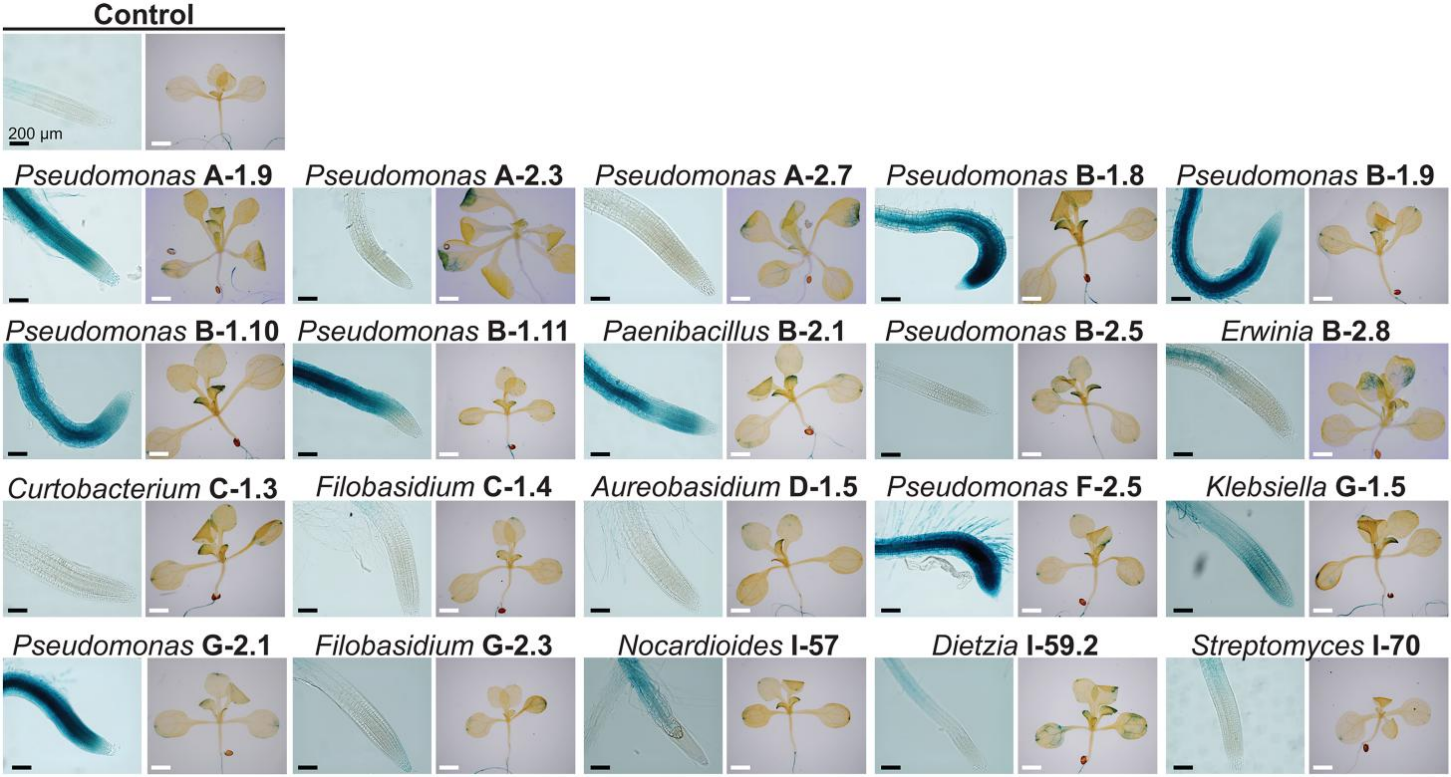
Effects of selected isolates on defense-related jasmonic acid gene expression in Arabidopsis. Four-day-old transgenic seedlings expressing the jasmonic acid-inducible *JAZ1/TIFY10A::GFP:uidA* gene marker were transferred to 0.2× MS medium inoculated with the different isolates to analyze activation of the JA signaling pathway 7 d after the interaction (at least 10 seedlings per treatment were analyzed).

JA is one of the main phytohormones orchestrating the plant defense response against biotic stressors with developmental programs (Raya-González *et al*., 2012; Carvalhais *et al*., 2017). Sensing of plant growth-promoting (PGP) microorganisms may result in triggering JA-mediated induced systemic resistance (ISR) and the recruitment of beneficial microbes through JA signaling (Carvalhais *et al*., 2017). Therefore, we analyzed the expression of the JA-responsive *Jasmonate-zim domain protein 1* (*JAZ1*) gene as an indicator of pathway activation in *JAZ1/TIFY10A::GFP:uidA A. thaliana* transgenic seedlings, directly or indirectly interacting with the 20 strains selected for their activity against necrotrophic phytopathogens and PGP properties. Histochemical GUS activity assays in the co-cultured seedlings indicated that *Pseudomonas* spp. A-1.9, A-2.3, B-1.8, B-1.11, and F-2.5, *Paenibacillus* sp. B-2.1, and *Streptomyces* I-70 induced an exacerbated gene expression in shoot tissues and in the apical region of primary roots, from the xylem to the external cell layers, which coincides with developmental shifts such as root hair differentiation, shortness of the meristematic zone, and thickening of roots (Fig. 7). The bacterial isolates *Pseudomonas* spp. A-2.3, A-2.7, and B-2.5 and *Curtobacterium* sp. C-1.3, and the yeasts *Filobasidium* spp. C-1.4 and G-2.3, and *Aerobasidium* sp. D-1.5 induced the expression of the JA-responsive gene only in the shoot system (Fig. 7), probably indicating the activation of an ISR response. The isolates *Erwinia* sp. B-2.8, *Klebsiella* sp. G-1.5, *Dietzia* sp. I-57, *Nocardioides* sp. I-59.2, and *Streptomyces* I-70 induced the systemic activation of the JA-responsive pathway with a moderated root expression and shoot-induced signal of the gene marker (Fig. 7), which could be differentially related to a PGP or to pathogenic activity. The volatiles emitted by *Pseudomonas* spp. A-1.9, A-2.3, A-2.7, B-1.8, B-1.10, B-2.5, and G-2.1, *Paenibacillus* sp. B-2.1, *Curtobacterium* sp. C-1.3, *Klebsiella* sp. G-1.5, and *Dietzia* sp. I-59.2 increased *JAZ1* gene expression (Supplementary Fig. S3), indicating a possible role as airborne bacterial signals in interactions with plants.

## Discussion

In angiosperms, floral structures constitute the first interphase in plant–pollinator interactions, where nectar represents the main nutritional reward (Vannette, 2020). Floral nectar harbors specialized microbiota that could impact both plant and pollinator health (Vannette *et al*., 2013; Martin *et al*., 2022; Álvarez-Pérez *et al*., 2024). However, beneficial traits of the nectar microbiota to antagonize plant and pollinator pathogens and promote plant fitness at early developmental stages have been scarcely explored. Here, we reported for the first time the composition of the nectar-inhabiting bacterial and fungal communities, and some functions of their culturable fraction, in a crop with agronomical, ecological, and evolutionary importance, the avocado *P. americana* Mill.

Ubiquitous microbes reach nectar via wind, rain, and animal transport. However, only a few taxa are able to survive UV radiation, the presence of antimicrobial compounds, low nitrogen availability, and osmotic stress that characterize nectar as a highly selective ecological niche (Pozo *et al*., 2011). A low microbial diversity in nectar community composition suggests that dominant taxa could be particularly important for plant health (Good *et al*., 2014; Jacquemyn *et al*., 2021). In hexose-rich nectars, specialist yeasts of the genus *Metschnikowia* predominate, followed by the generalist *Aureobasidium* and *Cryptococcus*, while the most common bacteria belong to the Enterobacteriaceae family and to the genera *Acinetobacter* and *Bacillus* (Pozo *et al*., 2011; Vannette, 2020; Mueller *et al*., 2023; Álvarez-Pérez *et al*., 2024). Here, the high relative abundance of *Acinetobacter*, *Pseudomonas*, and *Vishniacozyma* in avocado nectar was consistent with that reported in other nectars, but *Bacillus*, *Metschnikowia*, and *Cryptococcus* were not included among the most abundant taxa in the analyzed samples, while this was the first report of *Protomyces* as the dominant taxon in nectar (Fig. 1). These compositional differences in nectar microbial communities are probably due to the chemical composition of avocado nectar and the species that pollinate its flowers (de Vega *et al*., 2009). Perseitol content in avocado nectar could be inhibiting the germination of *Bacillus* spores (Rizk *et al*., 1989), while having a nectar rich in sucrose with low fructose and glucose content is not optimal for the development of an alcohol-fermenting yeast incapable of hydrolyzing sucrose, such as *Metschnikowia* (Vicente *et al*., 2020; Torrellas *et al*., 2023). The case of *Protomyces*, a little-researched phytopathogen in floral nectar with the ability to assimilate sucrose and which causes hyperplasia and formation of galls in the veins of leaves, petioles, peduncles, stems, and fruits (Čadež *et al*., 2021; Leharwan *et al*., 2021), is interesting, since there are no reports of this genus as a causal agent of diseases in avocado (González-Santarosa *et al*., 2014), suggesting that avocado nectar could act as a reservoir for this phytopathogen. Finally, even the most abundant genera are likely to benefit from each other, since not all can use sucrose as a carbon source. For example, *Acinetobacter* spp. and *Filobasidium* spp. are both non-saccharolytic. Whilst the bacteria have *n*-alkanes, the yeast degrades higher fatty acid esters and terpenoids to promote the aroma and flavor in fruits (Wang *et al*., 2023; Wu *et al*., 2023). They both probably thrive on avocado nectar at the expense of *n*-alkanes, VOCs, and polyphenols that are synthesized to mediate interactions with insects and other microbes and provide protection against UV radiation, as reported in other plant species (Bartwal *et al*., 2013). On the other hand, *Aureobasidium* spp. and *Pseudomonas* spp. can assimilate a wide range of compounds, but the latter prefer organic acids and amino acids as a carbon source, rather than sugars (Rojo, 2010; Chi *et al*., 2022). *Pseudomonas* spp. could therefore benefit from the tricarboxylic acids produced by other microorganisms that inhabit the nectar (Anderson and Guerra, 1985).

Our data indicated that 20 out of 43 tested isolates were active against *C. gloeosporioides*, *P. cinnamomi*, and entomopathogens in dual culture, whilst their emitted volatiles promoted plant development (Figs 2–4). This reinforces the theory that the few microbial taxa present in nectar play important roles in plant fitness (Good *et al*., 2014) and demonstrates a high potential of nectar for as a source of beneficial microorganisms for agriculture. Recently, the antagonistic activity against *Colletotrichum* pathogens of several nectar microbes, isolated from blueberries, was described in *in vitro* assays, showing that the pathogen inhibition did not affect nectar consumption by honeybees (Rering *et al*., 2023). Here, most of tested isolates displayed a greater pathogen inhibition through direct contact than through volatile emission, which suggests that the inhibition of mycelial growth observed in dual culture assays could be due to the production of diffusible compounds and cell wall-degrading enzymes. *Pseudomonas* spp., *Paenibacillus* spp., *Klebsiella* spp., and *Aureobasidium* spp. produce phenazines, exophilins, lyamocins, and lytic enzymes such as proteases and chitinases that damage the fungal cell wall (Merín *et al*., 2014; Raza *et al*., 2015; Di Francesco *et al*., 2017; Jisha *et al*., 2018; Hsu *et al*., 2021; Zhang *et al*., 2022; Iqbal *et al*., 2023). This is supported by the microscopy images showing damage of the mycelium of the different pathogens when exposed to nectar isolates. Future analysis of the metabolomic and volatile profiles from our antagonistic microbial isolates, exposed to different artificial nectar formulations, should shed some light on these antifungal chemical compounds.

Molecular identification of the selected nectar isolates largely coincided with the genera that reached the highest relative abundances in the metabarcoding analysis (Fig. 1; Table 1). *Pseudomonas* was one of the most dominant genera of the community, with a relative abundance of 32.8%, and 50% of the bioactive microbial isolates belonging to this genus. Similarly, *Erwinia*, *Klebsiella*, and *Filobasidium* were among the taxa with the highest relative abundance in the nectar microbial community, and were also retrieved in the culturable fraction. Exceptions included *Paenibacillus* sp. B-2.1, *Curtobacterium* sp. C-1.3, *Dietzia* I-57, *Nocardioides* I-59.2, and *Streptomyces* I-70, which were not detected among the most abundant taxa in avocado nectar, and *Acinetobacter*, the bacterial genus with the highest relative abundance in the community, although none of the 20 isolates selected for molecular identification corresponded to this taxon. This could be because *Acinetobacter* spp. from avocado nectar do not present antagonistic activity against *C. gloeosporioides* and *P. cinnamomi*, which was our main selection criterion. However, since antagonistic activity of *Acinetobacter* spp. strains has been reported against several *Colletotrichum* and *Phytophthora* species, this warrants future efforts to retrieve *Acinetobacter* isolates and investigate their potential bioactivity (Syed-Ab-Rahman *et al*., 2018; Patel *et al*., 2019; Matos *et al*., 2024).

Regarding the effect of nectar microorganisms on plant fitness, microbial volatile and direct contact interactions with our model plant showed that volatile emission by all the evaluated strains promoted root branching and biomass accumulation, while the accumulation of some diffusible microbial metabolites led to activation of the JA defense pathway (Figs 5–7). Volatile and diffusible compounds from *Pseudomonas* spp., *Klebsiella* spp., *Dietzia* spp., *Streptomyces* spp., and *Aureobasidium* spp. have been explored for their role in promoting plant development. Benzyl alcohol, 2-phenylethyl alcohol, 3-(methyl-thio)-1-propanol, trimethylamine, 3-hydroxy-2-butanone (acetoin), 2,3-butanediol, and 2-pentylfuranare are some examples of VOCs produced by these genera that promote nutrient availability and root development (Camarena-Pozos *et al*., 2019; Jones *et al*., 2019; Ravelo-Ortega *et al*., 2023), and should be further studied for their effects on avocado development.

In this context, the fact that all tested isolates emitted VOCs with plant growth-promoting activity (Fig. 5), despite belonging to different taxa, is noteworthy. However, different species related to the isolates described here have been shown to produce widely known plant growth-promoting volatiles; for example, *Paenibacillus polymyxa* emits acetoin and 2,3-butanediol, the best-known growth-promoting VOCs (Ryu *et al*., 2003; Lee *et al*., 2012). *Pseudomonas* spp., *Erwinia amylovora*, and *Curtobacterium flaccumfacien* also produce 2,3-butanediol (Cho *et al*., 2008; Santoro *et al*., 2011; Groenhagen *et al*., 2013; Parmagnani *et al*., 2023; Gaudioso *et al*., 2025), while trimethylamine produced by *Streptomyces venezuelae* has recently been shown to increase the rate of photosynthesis, thus contributing to improve plant health and growth (Li *et al*., 2025). On the other hand, yeasts such as *Filobasidium* and *Aerobasidium* often produce dimethyl disulfide, which promotes root system development (Groenhagen *et al*., 2013; Meldau *et al*., 2013). Although the VOCs released by *Dietzia* spp. and *Nocardioides* spp. are less explored, they produce ketones, alkanes, lipids, alcohols, and sulfides, compounds similar to those mentioned for their activity as promoters of plant development (Biçer *et al*., 2021; Widada *et al*., 2021). Since several reports provide evidence of how a single VOC, even at low concentrations, can modulate plant growth through different hormone-related pathways (Garnica-Vergara *et al*., 2016; Raya-González *et al*., 2017), further studies are needed to determine the molecular mechanisms underlying the plant growth promotion by avocado nectar isolates.

The activation of JA signaling by nectar microbial isolates is of particular interest, since it could, in addition to coordinating the defense against fungi, drive nectar secretion and the plant response against herbivores and their mechanical damage (Heil *et al*., 2012; Millán-Cañongo *et al*., 2014; Wang *et al*., 2021; López-García *et al*., 2023). These JA-inducing properties are especially striking in terms of ecological interactions, since some pathogenic bacteria can move into the vascular system of the host plants through the floral nectaries, for example *Erwinia tracheiphila*, the causative agent of wilt disease in cucumber (Sasu *et al*., 2010). A previous report described how the strawberry rhizosphere strain *Streptomyces globisporus* SP6C4 is able to translocate endophytically from roots to flowers and back to the roots, and to antagonize the gray mold pathogen *Botrytis cinerea* whilst reducing honeybee mortality caused by *P. larvae* and *Serratia marcescens* (Kim *et al*., 2019). These results support the idea that the rhizosphere, the anthosphere, and their associated microbiota impact the plant ecosphere, including the health of pollinators.

It is important to note that avocado nectar communities may also possess other functional attributes that are not explored here. According to several reports, some isolates belonging to the genera *Dietzia* and *Nocardioides* could protect plants against salt stress, heavy metals, and other air pollutants (Bharti *et al*., 2016; Ma *et al*., 2023; Shahid *et al*., 2024). Some species related to our identified isolates have traditionally been reported as phytopathogens, such as *Curtobacterium flaccumfaciens* (López-Ramírez *et al*., 2022; Tokmakova *et al*., 2024) and nectar specialists *Erwinia* (Pozo *et al*., 2011; Jacquemyn *et al*., 2013; Vannette, 2020). However, our data indicated that volatile and diffusible compounds of *Erwinia* sp. B-2.8 and *Curtobacterium* sp. C-1.3 promoted root system development in *A. thaliana* seedlings. Further genomic insights into the most promising plant growth-promoting strains will allow us to gain a deeper understating of the mechanisms underpinning their bioactivity.

Collectively, our results show that avocado nectar harbors a competitive specialized microbiota with bioactivity either as a phytostimulant, mostly through the emission of volatile compounds, or as contributors to plant health, by antagonizing pathogens and activating the JA defense pathway. Our findings suggest that the nectar microbiota could directly impact plant fitness and contribute to the health of its pollinators. Future work will focus on exploring the ecological significance of these nectar microbes in the avocado–pollinator interactions, by evaluating their attractiveness to floral visitors and their ability to disperse in the environment and colonize different ecological niches.

## Supplementary Material

erag038_Supplementary_Data

## Data Availability

The strain sequences were deposited in GenBank (accession nos PV366450–PV366466 and PV366592–PV366594). Data derived from bacterial and fungal sequencing were deposited in the Sequence Read Archive of NCBI under accession PRJNA1236352 (https://www.ncbi.nlm.nih.gov/sra/PRJNA1236352).

## Supplementary data

The following supplementary data are available at *JXB* online.

Table S1. List of primers used to create the 16S and ITS2 gene libraries to characterize the nectar microbial communities

Table S2. Classification of isolates into morphotypes according to their growth in LB medium and their Gram staining.

Fig. S1. Heatmap showing the most abundant bacterial ASVs.

Fig. S2. Heatmap showing the abundance of the fungal ASVs.

Fig. S3. Quantitative analysis of anthracnose lesions in avocado fruits.
