# Supplementary material for: The microbiota of avocado floral nectar inhibits pathogens and improves plant fitness
Source: J Exp Bot. 2026 Jan 23;77(8):2549–64. doi: 10.1093/jxb/erag038 (PMC13080368; doi:10.1093/jxb/erag038)
Supplement: erag038_Supplementary_Data [file erag038_supplementary_data.pdf]

Table S1. List of primers used to create the 16S and ITS2 gene libraries to characterize the nectar microbial communities.

ID	Sequence	Direction
16S515F	GTGYCAGCMGCCGCGGCCGCGGTAA	Forward
16S806R	GGACTACNVGGGGGTWTCTAAT	Reverse
ITS3NGS1	CTAGACTCGTCATCGATGAAGAACGCAG	Forward
ITS3NGS2	CTAGACTCGTCAACGATGAAGAACGCAG	Forward
ITS3NGS3	CTAGACTCGTCACCGATGAAGAACGCAG	Forward
ITS3NGS5	CTAGACTCGTCATCGATGAAGAACGTGG	Forward
ITS4NG	TCCTCCGCTTATTATTGATATGC	Reverse

Table S2. Classification of isolates into morphotypes according to their growth in LB medium and their Gram staining.

Morphotype	Colonial morphology	Microscopic analysis
**A**	Creamy yellow colonies	Gram-negative rods
**B**	White, smooth colonies	Gram-negative rods
**C**	Beige, smooth colonies	Yeast
**D**	Yellowish, smooth colonies	Yeast
**E**	Yellowish, smooth colonies	Gram positive coccobacilli
**F**	White, mucoid colonies	Gram-positive filaments
**G**	Creamy smooth white colonies	Yeast
**H**	White, smooth, mucoid colonies	Gram-positive filaments
**I**	White, rough, mucoid colonies	Gram-positive filaments

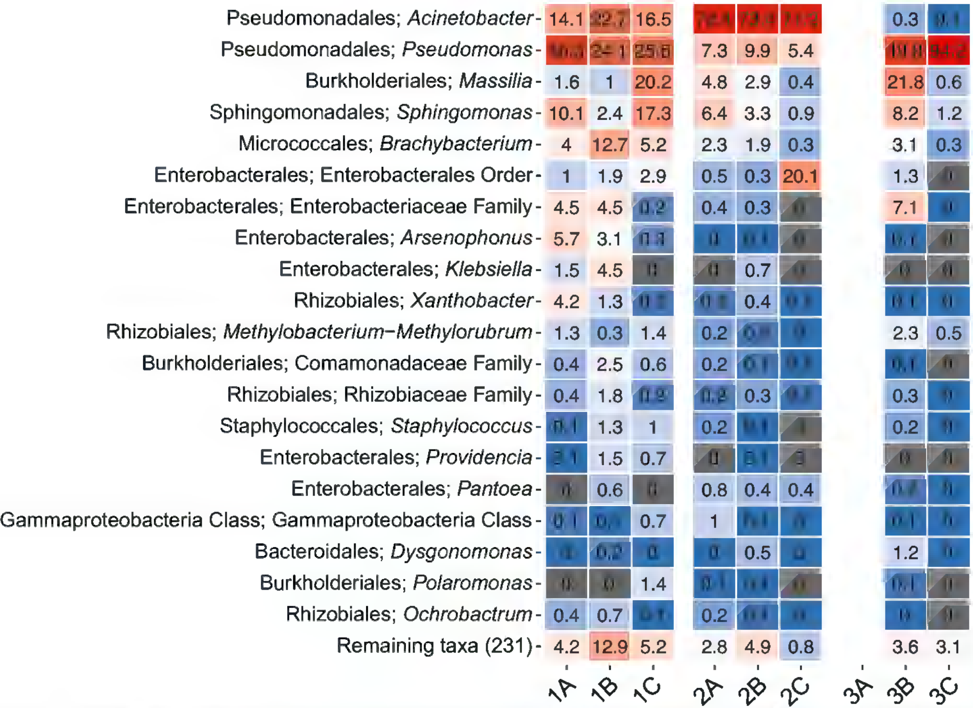

Fig. S1. Heatmap showing the most abundant bacterial ASVs. The color scale (from blue to red) indicates the relative abundance of the assigned ASVs at genus-level taxonomy by 16S rRNA gene sequencing. Note that sample 3A was removed from the analysis.

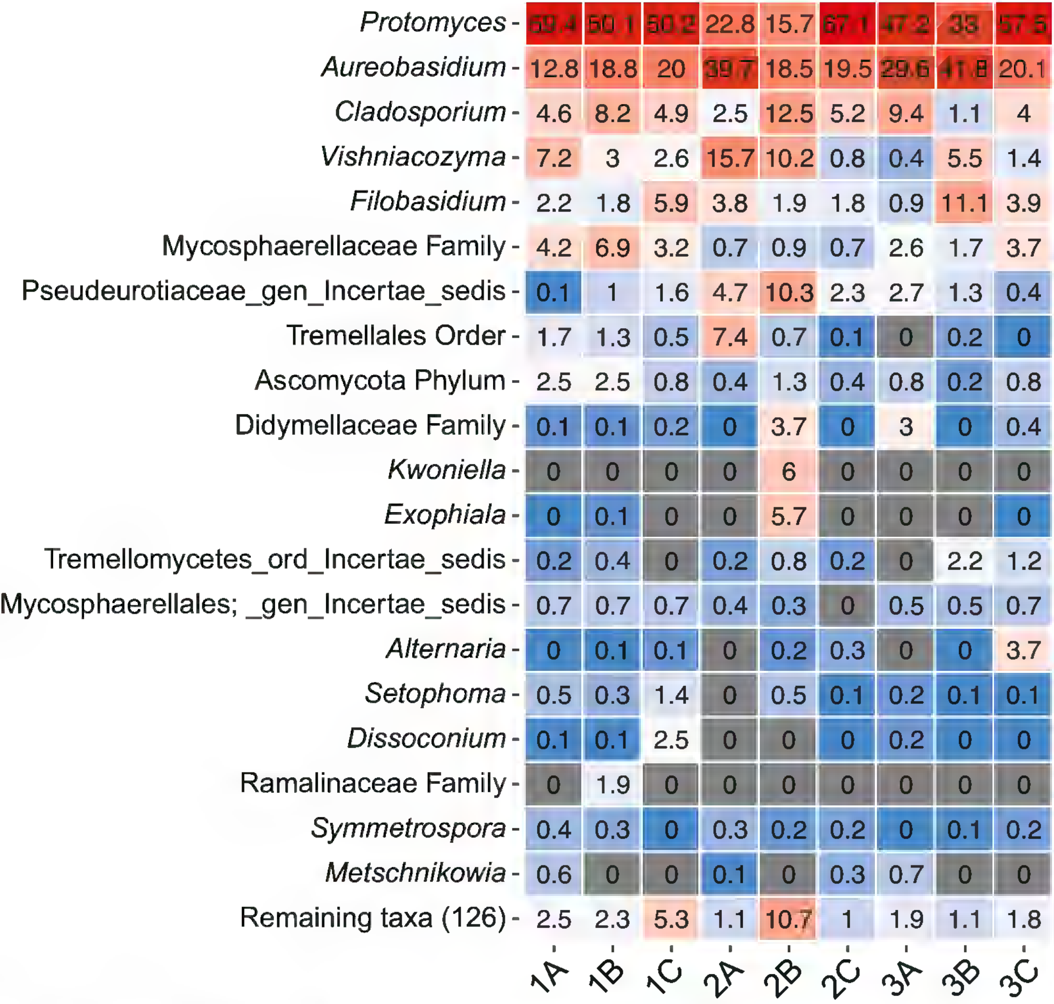

Fig. S2. Heatmap showing the abundance of the fungal ASVs. The color scale (from blue to red) indicates the relative abundance of the assigned ASVs at genus-level taxonomy (when possible) by ITS2 barcode sequencing.

A)

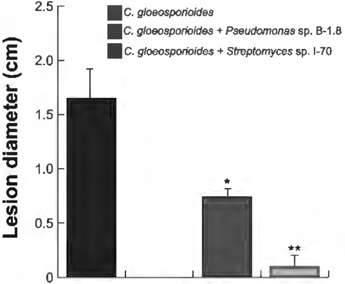

B)

	Lesion inhibition (%)
***Pseudomonas* sp. B-1.8**	55.26 ± 4.04
***Streptomyces* sp. I-70**	93.85 ± 6.15

Fig. S3. *Pseudomonas* sp. B-1.8 and *Streptomyces* sp. I-70 reduced the damage caused by *C. gloeosporioides* in avocado fruit. Images of fruit infected with the phytopathogen were processed using the ImageJ2/Fiji software to determine the diameter of lesions (A). Measurements were used to calculate the percentage of lesion inhibition by the bacteria (B). Values represent the mean ± standard error, and asterisks indicate a significant difference with a *P* value ≤ 0.05. Assays were performed in quadruplicate with similar results.
